# 13-*cis*-retinoic acid re-differentiation therapy and recombinant human thyrotropin-aided radioiodine treatment of non-Functional metastatic thyroid cancer: a single-center, 53-patient phase 2 study

**DOI:** 10.1186/1756-6614-2-8

**Published:** 2009-08-01

**Authors:** Daria Handkiewicz-Junak, Jozef Roskosz, Kornelia Hasse-Lazar, Sylwia Szpak-Ulczok, Zbigniew Puch, Aleksandra Kukulska, Tomasz Olczyk, Andrzej Piela, Ewa Paliczka-Cieslik, Barbara Jarzab

**Affiliations:** 1 Department of Nuclear Medicine, Maria Sklodowska-Curie Memorial Institute, Gliwice Branch, Wybrzeza Armii Krajowej 14, 44-100 Gliwice, Poland; 2 Department of Endocrine Oncology, Maria Sklodowska-Curie Memorial Institute, Gliwice Branch, Wybrzeza Armii Krajowej 14, 44-100 Gliwice, Poland; 3 Department of Surgical Oncology, Maria Sklodowska-Curie Memorial Institute, Gliwice Branch, Wybrzeza Armii Krajowej 14, 44-100 Gliwice, Poland

## Abstract

In 30–50% of patients with metastatic non-medullary thyroid cancer the metastases are not radioiodine-avid and so there is no effective treatment. Retinoids have demonstrated inhibition of thyroid tumor growth and induction of radioiodine uptake. The aim of our study was to assess benefits of the retinoic acid (RA) treatment to re-differentiate non-functional NMTC metastases.

**Patients and Methods:**

In this prospective study, 53 patients with radioiodine non avid metastatic disease (45) or hyperthyroglobulinemia (8) were treated with 13-cis-retinoic acid (13-CRA) [1.0 mg/kg/day over 1st week and then 1.5 mg/kg] for six weeks prior to I-131 treatment performed under rhTSH stimulation. The re-differentiating effect of RA was evaluated by serum thyroglobulin (Tg) monitoring before and after cessation of RA treatment and by qualitative analysis of iodine uptake on the post-therapeutic whole body scan (rxWBS).

**Results:**

13-CRA induced radioiodine uptake in 9 (17%) of patients. In the univariate analysis neither the patient's gender, age, tumor histopathology, uptake in thyroid bed nor time since thyroid cancer diagnosis was associated with results of rxWBS.

41 (77%) patients were evaluable for Tg response before and after to 13-CRA treatment. There was a statistically significant increase in median Tg level (60 v. 90 ng/ml, p < 0.05). There was no difference in Tg increase between scintigraphic responders and non-responders.

13-CRA and RIT was repeated at least once in 8 of 9 scintigraphic responders. None of them showed tumor regression by radiological imaging within 12 months after the first treatment, 4/9 (44%) of them had disease progression.

13-CRA treatment was well-tolerated. All but one patient complained of at least one side effect the most prevalent being lip dryness (98%). All side effects were transient and resolved within 2 weeks after 13-CRA cessation.

**Conclusion:**

Our results show that in patients with non-functional metastases from NMTC, 13-CRA is able to exert some re-differentiation effect by induction of radioiodine uptake in <20% of patients and increase of Tg serum level in about 30% of them. Nevertheless, this does not transfer into clinical benefit as it neither induces measurable tumor response nor prevents disease progression.

## Introduction

Soon after the introduction of 131-iodine (131-I) for treatment of thyroid cancer deriving from follicular cells (non-medullary thyroid carcinoma, NMTC) [1] it became evident that at least 30% of these tumors fail to concentrate radioiodine even after thyroid hormone withdrawal (THW) [2]. Over the ensuing decades, radioiodine non-avid thyroid cancer has remained a therapeutic challenge. Patients with non-functional thyroid cancer foci continue to have a much worse prognosis than their counterparts with 131-I uptake [3,4], and lack therapeutic options. Metastasectomy is useful solely in occasional patients with solitary, mainly bone lesions [5], while external radiation therapy can only palliate metastatic symptoms [6]. Conventional chemotherapy, i.e., doxorubicin alone or with cisplatin, is very toxic and provides merely a <20% rate of mostly very transient, partial responses [7].

In recent years, research has focused on targeted approaches addressing the pathological characteristics of radioiodine non-avid thyroid carcinoma. It has been known that inability to take up 131-I is associated with poor differentiation and increased tumor grade [3]. However, the molecular basis of loss of function is not well known. Thus far, *p53 *mutation is the only genetic change clearly shown to correlate with poor differentiation or lacking differentiation. Other molecular events known to occur in NMTC like decrease or loss of sodium-iodine symporter (NIS) expression or of other thyroid-specific molecules (5'-DI [type I iodothyronine-5'-deiodinase], TSH receptor, pendrin) are rather consequences of earlier molecular processes. Some recent reports show a significant correlation between the presence of initiating BRAF mutation and poorer outcome of DTC (differentiated thyroid cancer) or loss of function of DTC metastases [8]. However, BRAF mutation is a rather frequent initiating event in DTC (40–60% of all cases), while poor differentiation and progressive course are observed only in a significantly smaller fraction (circa 10–15%) of them. Thus, molecular processes linking BRAF mutation with still unknown events leading to increased proliferation and decreased function in this subset need to be uncovered.

Retinoids are a family of substances including the biologically active vitamin A metabolites, the retinoic acids (RA), that play an important role in morphogenesis, differentiation and proliferation of many cell types [9,10]. In NMTC tumor cells, experimental data demonstrate that retinoids can inhibit tumor growth and induce radioiodine uptake [11]. Additionally, retinoids exert several re-differentiating effects: induction of 5'-DI [12,13] increased expression of NIS mRNA [14] and of the differentiation marker, alkaline phosphatase, or decreased expression of CD97, which is highly expressed in anaplastic thyroid carcinoma [15], as well as stimulation of intercellular adhesion molecule-1 synthesis [16]. Although leading to partial rather than complete thyroid cancer cell re-differentiation, these effects have been hoped to render radioiodine uptake and enable treatment of NMTC foci [17-22]. The promising experimental results and their potential therapeutic impact prompted a number of centers including ours to initiate clinical studies to evaluate the effectiveness of *13-cis*-retinoic acid (13-CRA) as re-differentiation therapy (RDT) in patients with advanced radioiodine non-avid thyroid cancer. In contrast to other investigators, we performed the radioiodine therapy under recombinant human TSH (rhTSH) stimulation rather than under thyroid hormone withdrawal (THW). Our rationale for doing so was twofold. Firstly, we wished to avoid possible additive or synergistic toxicity of high-dose RA and of hypothyroidism secondary to THW, which have potentially overlapping symptoms, namely, depression, headache, increased lipidaemia, dry skin or/and mucosa, and musculo-articular pain [19]. Secondly, we sought to compare concentrations of serum thyroglobulin (Tg) as the marker of thyroid cancer differentiation, before and after RA therapy without the confounding factor of weeks-long TSH elevation.

## Method

### Study Design and Ethical Considerations

**Figure 1:**
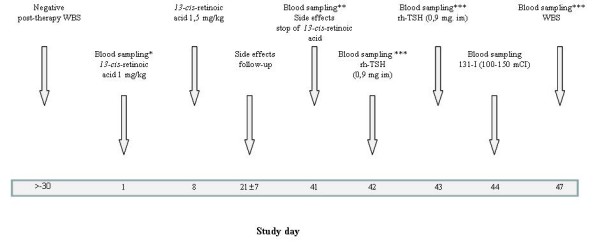
**Study design**. Radioiodine non-avid disease was confirmed by a negative post-therapy whole-body scan plus radiological imaging. Study day scale is not drawn proportionally. RAI, radioiodine treatment; rxWBS, post-therapy whole-body scan (* TSH, Tg, rTg, morphology, transaminase, cholesterol, creatinine. ** morphology, transaminase, cholesterol, creatinine, in 20 patients also TSH, Tg, rTg. *** TSH, Tg, rTg)

This was a prospective phase II observational study. The protocol (Figure 1) was approved by the local ethics committee. All patients were informed of the nature, aim and potential risks of the study therapy and provided written consent before the treatment was initiated.

The primary endpoint was the: (1) the qualitative effect of *13*-CRA RDT on 131-I uptake in confirmed non-functional NMTC metastases (scintigraphic response) and (2) the quantitative effect of 13-CRA RD Ton thyroglobulin serum level during therapy course. Secondary endpoints comprised tolerance of 13-CRA RDT/rhTSH-aided radioiodine treatment (rhTSH RIT) and tumor response in scintigraphic responders.

Inclusion criteria were: (a) metastatic or locoregionally advanced, papillary, follicular or poorly differentiated thyroid cancer, both with stable or progressive course of the disease; (b) no radioiodine uptake in known metastatic thyroid cancer foci on the most recent post-therapy whole body scintigraphy (rxWBS) and (c) no prior retinoid RDT. Exclusion criteria included: (a) anaplastic thyroid cancer; (b) pregnancy or lactation; (c) life expectancy > 6 months; (d) serum transaminase (AST, ALT) < 80 mUI/ml; (e) serum total cholesterol < 360 mg/ml; and (f) serum triglycerides < 250 mg/%, (g) administration of iodine-containing drugs less than 3 months before planned radioiodine therapy.

### Study Population

The study population included all eligible patients (n = 53) who agreed to receive 13-CRA RDT and rhTSH RIT at our tertiary referral centre from 1999 – 2004. The group tended to be of mid-late middle age at study entry and was nearly 2/3 female; a similar proportion of patients had papillary histology at diagnosis (Table 1). All but one patient had been treated at least once with 131-I (up to five therapies) with a median accumulated activity of 6.66 GBq. In all but one case the therapies were performed under endogenous TSH stimulation (TSH > 25 mIU/ml). One patient was included who had not previously received radioiodine and hence had no prior rxWBS. This patient had been diagnosed with poorly-differentiated follicular cancer diffusely metastasized to bone and non-functional disease was confirmed by diagnostic WBC (dxWBS).

**Table 1:** Characteristics of 53 patients receiving *13-cis*-retinoic acid at start of re-differentiation therapy

Characteristic	Value
Female, n (%)	34 (64%)

Age at thyroid cancer diagnosis (years), median (range)	57 (15 – 69)

Primary histopathology, n (%)	
papillary	34 (64%)
follicular	17 (32%)
insular	2 (4%)

Localization of metastatic disease, n (%)*:	
lung	31 (58%)
locoregional disease	13 (25%)
hyperthyroglobulinaemia	8 (15%)
bones	6 (11%)
other	5 (9%)

Previous radioiodine treatments	
number, median (range)	2 (0–5)
cumulative activity – GBq; median (range)	6.66 (0.00–15.54])

Time from thyroid cancer diagnosis to *13*-CRA RDT, median (range)	54 (6–186) months

* the sum is bigger than 53 as in some patients there were more than one disease localization

Forty-five (85%) of the 53 patients suffered from overt disease, which in all instances was radiologically or ultrasonographically confirmed, or both. All cases of locoregional disease were confirmed by fine needle biopsy as well. As seen in Table 1, the most prevalent metastatic involvement was of the lung and of the neck lymph nodes, thyroid bed or both ("locoregional" sites). Metastases to multiple sites were detected in 10 (19%) patients. Median time from the disease diagnosis to diagnosis of metastatic disease was 30 months (range 0 – 171). In 13 patients metastases were diagnosed within a year after the diagnosis of thyroid cancer and in 5 of them more than 10 years after the primary diagnosis. Eight (15%) patients were diagnosed with hyperthyroglobulinaemia in range of 11–100 ng/ml during L4-therapy, which was presumed to reflect occult nonfunctional micrometastases (no radioiodine uptake nor abnormalities in radiological examinations).

Forty nine (92%) patients were previously treated with at least one RIT in our centre. Three (6%) were referred to us after being treated elsewhere and had their post-therapy scans sent to us for evaluation. As already mentioned, the negative post-radioiodine therapy scan was available in all but one patient. In 21 (42%) patients negative rxWBS was followed-up by negative diagnostic WBS. In no instance the most recent WBS did show any radioiodine uptake in any metastasis, but some physiological thyroid bed uptake was visible on rxWBS in 10 (19%) patients (Table 2) who were treated after total thyroidectomy. From 36 patients who were treated more than two times before 13-CRA RDT, in 32 there was no other uptake than in thyroid bed, and in 4 patients initially there was some very weak uptake in metastatic sites that was no more visible in subsequent treatments.

**Table 2:** Scintigraphic findings after 13-CRA therapy in 53 patients with radioiodine non-avid NMTC metastases

WBS after redifferentiation therapy	Last pre redifferentiation WBS			
	No uptake		Uptake in thyroid bed	
No scintigraphic response in metastases	35*	(66%)	8	(15%)

Scintigraphic response in metastases	8	(15%)	1	(2%)

Lung	5	(9%)	0	(0%)
Mediastinum	1	(2%)	1	(2%)
Lung and lymph node	1	(2%)	0	(0%)
Lung and bone	1	(2%)	0	(0%)

Persistent thyroid bed uptake in pre- and post-13-CRA RDT rxWBS	0	(0%)	1	(2%)

13-CRA, 13-cis-retinoic acid; RDT, re-differentiation therapy;

rxWBS, post-therapy whole-body scintigraphy

* in one patient there was only diagnostic WBC for comparison

For 23 patients in the present series, earlier results have been reported elsewhere [31]

### 13-CRA RDT

RDT with *13*-CRA (Roaccutan^®^, Roche AG, Germany) was started 6 weeks before radioiodine administration (Figure 1). The dosage was 1.0 mg/kg of body weight/day during the first week and if that dose was tolerated, 1.5 mg/kg/day for the next 5 weeks, during which time dose reduction to 1.0 mg/kg was allowed in case of drug intolerance.

### RIT

RIT was performed under exogenous stimulation with two consecutive daily intramuscular injections of 0.9 mg of rhTSH (Thyrogen^®^, Genzyme Corporation, Cambridge, MA) on study days 42 and 43. On study day 44, a fixed empirical therapeutic radioiodine activity of 3.70 GBq, (in 2 patient 5.55 GBq was applied) was given by oral capsule. When the first study rxWBS showed radioiodine uptake in one or more metastatic sites, the study treatments were repeated at 6-month intervals, until pathological uptake was lost on rxWBS due to cure or disease progression. When the initial study rxWBS was negative, the patient resumed follow-up and medical care in his/her community.

### Endpoint Assessment

The effect of 13-CRA RDT on 131-I uptake was evaluated through qualitative assessment of the study day 47 rxWBS for such uptake in sites of previously confirmed radioiodine non-avid metastases (based on "yes/no"answer). Three experienced nuclear medicine physicians (DHJ, JR, BJ) rated these sites for scintigraphic response, i.e., presence or absence of visual uptake. In case of discrepant ratings the scans were reevaluated by three physicians together. The patient was evaluated as scintigraphic responder when at least one focus of definite 131-I uptake was diagnosed concordantly with localization of known metastasis.

rxWBS was performed 3 days after the second rhTSH injection using a dual-head gamma camera (Siemens Multispect 2) with parallel high-energy collimators, for at least 140,000 counts or 20 minutes of acquisition. Single spot images of regions containing known or suspected metastases were taken for at least 80,000 counts.

Tg levels were compared in blood samples obtained within 7 days before the start of 13-CRA RDT, versus in those obtained on study day 42. Study day 42 sampling took place before the first rhTSH injection, which also was given that day. To place into context any effect of 13-CRA RDT on serum Tg concentrations, Tg levels in the study day 42 sample also were compared with those in the sample taken on study day 47, 3 days after the second and last rhTSH injection. To establish physiological variations in serum Tg concentration, this analyte was additionally measured one day before last 13-CRA application in 20 (38%) randomly chosen patients. The highest difference between these two measurements was 30%. Thus an increase of ≥30% over pre-RDT levels was chosen to denote a clinically relevant change in Tg and the patient was evaluated as biochemical responder when the rise in serum Tg exceeded 30%.

Tg was measured by immunofluorometric assay (Wallac Delfia) with functional sensivity of 1 ng/ml. Interference by anti-Tg antibodies was ruled out via Tg recovery testing performed simultaneously with the Tg assay. Recovery beneath our institutional cut-off of 70% was considered to denote such interference, and caused the patient to be excluded from further Tg analysis [23].

Side effects of 13-CRA RDT were assessed through structured questioning of patients and by caregiver observation after 3 weeks and at the end of therapy. Known potential symptoms of 13-CRA including depression, headache, vision problems, hearing loss, nosebleed, skin, dry, itching or peeling lips, skin, or other mucosa, and musculo-articular pain were specifically sought. Lipaemic effects and hepatotoxicity were assessed through measurement of serum cholesterol, triglyceride and transaminase levels in blood samples taken on days 1, 21 and 42. Additionally, patients were requested to spontaneously report any apparent side effects or health status changes throughout the study.

Tumor response to the study treatment was evaluated only in scintigraphic responders, by physical, scintigraphic and radiological (contrast enhanced computer tomography) examinations performed immediately after the first course of 13-CRA RDT/rhTSH RIT and after subsequent courses.

### Statistical Methods

A p value ≤ 0.05 was deemed significant. The chi-square test was used to compare frequencies in subgroups. Thyroglobulin measurements (non normal distribution) were compared with the Wilcoxon nonparametric matched pairs test. The univariate analysis was conducted to identify independent predictive factors for scintigraphic response. Statistical analyses were performed with the *Statistical for Windows *package (Statsoft Polska, Kraków, Poland).

## Results

### Scintigraphic Response

Forty-four (83%) of 53 patients had no uptake and 9 (17%) showed uptake in known metastatic sites (Table 2). In one of the scintigraphic responders the latest THW-aided rxWBS showed uptake in thyroid bed. One of ten patients with thyroid bed uptake on the latest THW-aided rxWBS retained such uptake on the study day 47 rxWBS (Table 2).

### Potential Predictive Factors for Scintigraphic Response

In univariate analysis, neither the patient's gender, age, tumor histopathology at diagnosis, 131I uptake in thyroid bed during the last pre study RIT or time since thyroid cancer diagnosis was statistically significantly associated with scintigraphic response (Table 3). Therefore no multivariate analysis of potential predictive factors for scintigraphic response was performed.

**Table 3:** Univariate analysis of potential predictive factors for scintigraphic response to 13-*cis*-retinoic acid therapy

**Variable**	Uptake in metastases (n = 9)	No uptake in metastases (n = 44)	*P*¶
Potential predictive factor			
**Sex**			Ns
Female (n = 34) vs.	7 (78%)	27 (61%)	

Male (n = 19)	2 (22%)	17 (39%)	

**Histology**			Ns
Papillary (n = 34) vs.	4 (44%)	30 (68%)	

Follicular (n = 17) vs.	4 (44%)	13 (30%)	

Insular (n = 2)	1 (2%)	1 (2%)	

Median age	54	59	Ns

Mean time since diagnosis of metastatic disease, years	5.3	3.5	Ns

Mean time since last 131-I therapy, months	19.4	15.3	Ns

Results of the last pre-redifferentiation post therapy WBS:			Ns
uptake in thyroid bed	1	9	
no uptake	8	35	

¶ NS, not significant (*P *> 0.05)

### Thyroglobulin response

Six (11%) of 53 patients were considered to have interference from anti-Tg antibodies based on Tg recovery <70%, and were excluded from the analysis of Tg response. In another 6 patients (11%), no evaluable pre-RDT blood sample was available due to 13-CRA RDT being started by the patient's local endocrinologist (n = 2) or due to the patient erroneously withdrawing thyroid hormone before 13-CRA (n = 4, in all of whom thyroid hormone was re-started on day 1 of 13-CRA RDT). Thyroglobulin levels at the first and last 13-CRA RDT application are depicted in additional file 1: thyroglobulin before and after 13-CRA RDT.

Thus, 41 (77%) patients were evaluable for Tg response to 13-CRA RDT. In this group, baseline serum Tg levels at the start of 13-CRA RDT ranged from 0.2 to 4079.0 ng/ml (median 60.0 ng/ml, mean ± SD, 637 ± 1227 ng/ml). The change between baseline and post-13-CRA RDT (i.e., day 42) Tg values was beneath the prospectively defined >30% threshold for clinical relevance in 28 (68%) of the 41 and was above that threshold in 13 (32%) (see additional file 1: thyroglobulin before and after 13-CRA RDT). In fact all of them exhibited the response of >50% of the initial level. Thyroglobulin level increased in 1 (17%) out of 6 evaluable patients with 13-CRA RDT-induced 131-I uptake and in 12 (34%) out of 35 without radioiodine uptake on rxWBS (p > 0.05).

**Figure 2:**
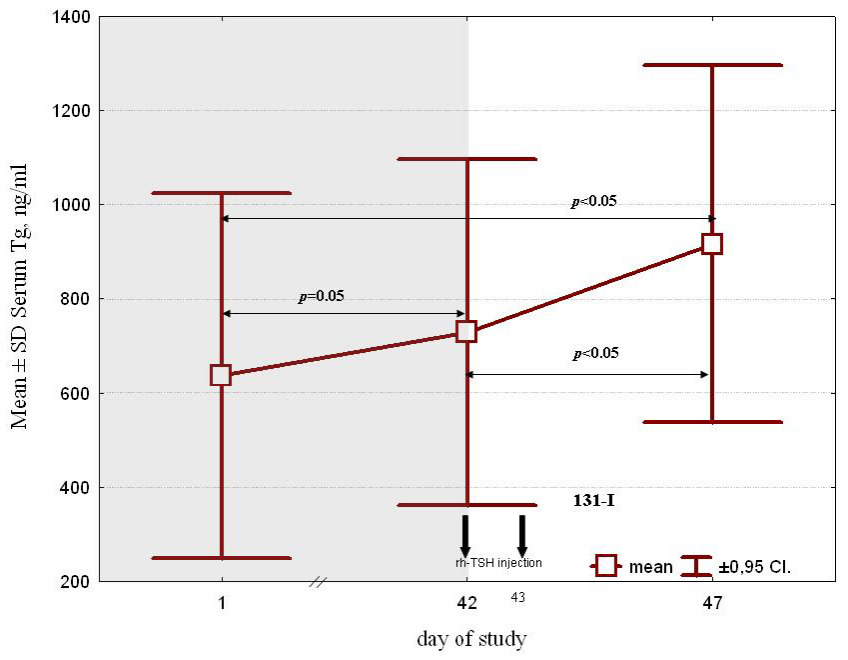
**Serum thyroglobulin changes during 13-*cis*-retinoic acid therapy and after administration of recombinant human thyroid-stimulating hormone therapy, 0.9 mg/day × 2**. Only patients (n = 41) showing no interference from anti-thyroglobulin antibodies, based on thyroglobulin recovery >70% were included. Hollow squares indicate the median value, while t bars indicate the 25% and 75% percentile. X-axis time scale is not exact.

The difference (n = 41) between the median pre- and post-13-CRA RDT Tg concentration was statistically significant. There was also a statistically significant difference between the mean Tg values before and after rhTSH stimulation (i.e., day 42 versus day 47 levels) (Figure 2).

In 7 patients (13% of evaluable patients, 54% of Tg responders), the increase in Tg level after 6 weeks of 13-CRA exceeded 75%. In Tg responders, the median baseline serum Tg levels (36 ng/ml) at the start of 13-CRA RDT was lower then in non-responders (94 ng 7 ml), but the difference was statistically insignificant.

### Side effects of 13-CRA and rhTSH aided radioiodine therapy

The first course of 13-CRA was well-tolerated. All patients completed the therapy, and only 5 (9%) had toxicity that necessitated reduction of the 13-CRA from 1.5 mg/kg/day to the starting dose of 1 mg/kg/day

All but one patient complained of at least one side effect, the most prevalent being lip dryness (52 patients, 98%). The other most frequent side effects included skin dryness (31 patients, 58%) most intense on face and hands, skin redness or mucosal dryness (21 patients, 39% each) or musculo-articular pain (15 patients, 28%). All side effects were transient and appeared to resolve within 2 weeks after 13-CRA cessation.

In biochemical evaluation following the first course of 13-CRA, a statistically significant increase from baseline to day 42 concentrations was noted in mean serum triglyceride, cholesterol and aspartate and alanine aminotransferase level (Table 4). Still, in none of the patients during follow-up clinically relevant sequel, such as stroke, hart attack or vain thrombosis, developed.

**Table 4:** Median biochemical response to their first course of 13-*cis*-retinoic acid

Analyte (n: number of evaluated patients)	Median			*P*, Day 42 vs. Day 1 Serum Concentrations
		
	Pre-treatment (Day 1)	Day 42	Absolute (Relative) Difference, Day 42 vs. Day 1	
Total cholesterol, mg/dL (n = 46)	208	220	20.5 (9.6%)	<0,05

Triglycerides, mg/dL (n = 41)	114	171	75.8 (54.1%)	<0,05

Aspartate aminotransferase, U/L (n = 42)	20	25	3.1 (13.4%)	<0,05

Alanine aminotransferase, U/L (n = 43)	18	23	2.7 (12.0%)	<0,05

rhTSH injections were well tolerated. However, of note, there was a case of a patient with a rapid progression of bone metastases diagnosed within days after rhTSH. The same effect was observed in this patient during the previous THW-based 131I therapy.

### Subsequent Study Treatments and Outcome in Scintigraphic Responders

The study treatments were repeated at least once (median 3 times, range 1 to 5 times) in 8 of 9 scintigraphic responders. One patient was lost from follow-up immediately after the first 131-I therapy. All subsequent courses of 13-CRA RDT/rhTSH RIT resulted in qualitatively similar radioiodine uptake in metastases on rxWBS as it was seen on the study day 47 rxWBS.

However, none of the 8 scintigraphic responders who received repeated therapy, showed metastatic tumor regression by radiological imaging. In fact, within 12 months after the first 13-CRA RDT/rhTSH RIT, 4/9 (44%) of the patients had disease progression.

## Discussion

The present single-center phase 2 trial of 6 weeks of 13-CRA RDT followed by rhTSH RIT in 53 patients with confirmed radioiodine non-avid advanced NMTC resulted in two main findings. Firstly, the study 13-CRA RDT regimen appeared to exert true re-differentiating effects, but only in a minority of patients. These effects were manifested in two ways: (1) the regimen induced 131-I uptake in previously non-functional metastases on rhTSH-aided rxWBS in 9 (17%) of 53 of patients, and (2) a significant serum Tg increase was observed in 13 of 41, or 32% of evaluable patients. Observations from other clinical trials [20-22] are not comparable with our study, and are, indeed, difficult to interpret, because of these trials' use of THW and their lengthier interval between measurements, which were conducted between RITs, i.e, months apart. To our knowledge only one study [21] showed increase in Tg concentration during and L-thyroxine therapy. However, patients population was rather small. The change in Tg serum level was measured after 1 month of therapy with 13-CRA, as at that point patients withdrew L-thyroxine therapy. Higher than 30% increase in Tg serum level was observed in 6/10 (60%) of evaluable patients in that study (extracted from the raw data in manuscript). In our study such increase was observed in 32% of patients and for our evaluable patient population as a whole (n = 41), the increase in mean Tg value over the course of 13-CRA RDT was statistically significant. Because of our avoidance of weeks-long THW and the resultant confounding factor of TSH elevation and because of the short, 6-week interval between measurements, Tg increases over the course of RDT seen in our study are likely to represent true re-differentiation effects rather than tumor growth. Laboratory explanation for this observation is somehow controversial. There are some experimental evidence for the phenomenon of a RA-induced increase in Tg mRNA. Kurebayashi et al. [24] showed an increase of Tg mRNA in human poorly differentiated papillary thyroid carcinoma cell line (KTC-1) after incubation with 1 μmol/l all-trans-retinoic acid for 24 hours; however, others performing similar experiments have reported unchanged Tg mRNA expression [25].

With respect to incidence of scintigraphic response our findings are less favorable than those of several previously published studies. Grunwald et al. [18] reported markedly increased uptake after 13-CRA RDT in 2 (17%) of 12 treated patients and in another 3 only a faint uptake. In another trial qualitatively assessed radioiodine uptake was increased in 9 (45%) of 20 patients [19]. However, in the later study only 4 patients had completely negative scan before 13-CRA RDT. In our study we observed induction of radioiodine uptake in 17% of treated patients.

Our trial's second main finding, and its observation of greatest clinical relevance, is that the study regimen's re-differentiation effects appear to be accompanied by little if any clinical benefit. 13-CRA RDT/rhTSH RIT was repeated in 8 of 9 scintigraphic responders, and none had complete or partial response and 44% had progression within the subsequent 12 months.

Our findings regarding frequency and magnitude of tumor response are far less favorable than those seen in the pooled German data [22], in which tumor size decreased in 6 (12%) evaluable patients, did not change in 22 (44%), and increased in 9 (18%). However, in that study 131-I therapy was performed under endogenous TSH stimulation and patients with decreasing, insufficient but still present radioiodine as well as complete absence of radioiodine uptake were included into the study. This could result in better prognosis for this group of patients. Data of Courbon et al. [21], where no partial responses were observed and 45% suffered from disease progression, more closely resemble our negative findings. We speculate that two factors could account for the poor clinical outcome of our scintigraphic responders. Firstly, re-differentiation effect may have been insufficient to obtain adequate 131-I retention in metastatic tumor cells. Secondly, the particularly advanced and aggressive tumors, seen in patients included in our study, may be more radio-resistant than most differentiated thyroid carcinomas. It must be kept in mind that induction of 131-I uptake by a tumor cell is only a first step in successful radioiodine treatment: the cell must be exposed to the radiation for a sufficient length of time, and must be susceptible to the cytotoxic effect of radiation.

Of note, our study differs from the other 13-CRA RDT trials discussed above in two respects. Firstly, radioiodine therapy was applied not after THW but after rhTSH stimulation. Secondly, our study included no patient with weak or equivocal pre-RDT metastatic uptake; in all cases, the most recent pre-RDT rxWBS was completely negative for metastatic uptake (albeit 10 of our 53 patients had some faint thyroid bed uptake on those scans). We are aware of single reports indicating on the better radioiodine uptake after THW than after rhTSH [26,27]. However, in the vast majority of cases of rhTSH-aided treatment of advanced differentiated thyroid cancer reported to date, the 131I uptake has been adequate [28,29]. Our own experience with rhTSH aided 131-I treatment of functional thyroid cancer metastases is good [29]. Only 1 (3%) of 31 patients with functional metastases lost radioiodine uptake during rhTSH-aided treatment (31), a loss that was rather related to disease progression than to the stimulation method. Therefore, in our opinion, the results of the present study argue that at least in patients with absent rather than greatly diminished function pre-RDT, 13-CRA does not influence radioiodine uptake in the clinically relevant manner.

A limitation of the present study was that we evaluated 12 months' clinical outcome only in a small fraction of patients, the 8 of 9 scintigraphic responders who received subsequent study treatments. For ethical and practical reasons, we did not ask scintigraphic non-responders, (many of whom were in poor general condition, lived relatively far from our tertiary referral center, or both) to return for follow-up. Given the paucity of published clinical data on direct cytotoxic effects of RAs on NMTC, we believe it unlikely that patients experienced clinical benefit of 13-CRA RDT in the absence of scintigraphic response.

Study regimen with 13-CRA was relatively non-toxic: side effects resolved within 2 weeks of 13-CRA discontinuation and were generally mild. All patients completed therapy and 48 of 53, or 91%, tolerated escalation in the second week of therapy from the starting dose of 1 mg/kg of body weight/day to 1.5 mg/kg/day. However, side effects were nearly universal: 52 of 53, or 98% of patients had at least one symptom. Mean serum total cholesterol and aminotransferase levels rose significantly despite the absence of THW and hence, hypothyroidism. Nevertheless, this was of no clinical importance as in none of the patients during study or follow-up symptomatic stroke, heart attack, vein thrombosis or liver disease developed. Simon et al [19] reported side effects in 50% of patients treated with 13-CRA, mainly, skin reaction like in our study. There was also an increase in triglycerides concentration in their study, however, statistically not significant (data extracted from the raw data in the original manuscript).

## Conclusion

Our and other studies suggest that, especially in patients with non functional NMTC metastases, 13-CRA induces uptake in only a small minority of patients and produces clinical benefit in few if any of those individuals. Further investigation of RDT should focus on new RA derivatives and new adjuvant re-differentiating and radio-sensitizing strategies. Additionally, better predictive factors should be identified for scintigraphic and clinical response. In that regard, *in vitro *work [10,14,30] suggests that thyroid cancer re-differentiation with RA requires both intact RA receptor (RAR) expression and pathways in target cells: to respond to retinoid therapy with suppressed proliferation and increased apoptosis, a cell must express the RAR isoforms, RARβ and RXRγ. Bexarotene, a retinoid × receptor activator, raised some new hopes of effective redifferentiation therapy of NMTC but clinical experiences were rather disappointing as there was a poor matching between radioiodine uptake and metastatic foci visible in computer tomography [31]. Finally, targeted therapeutic strategies other than RA RDT, e.g., kinase inhibitors, merit considerable attention [32,33].

## Competing interests

Dr. Handkiewicz-Junak and Dr. Jarzab declare receiving consulting and lecture fees from Genzyme. No other potential conflict of interest relevant to this article was reported.

## Authors' contributions

DHJ conceived the research work, coordinated the data collection and prepared the manuscript; JR reviewed patients data, KHL reviewed patients data, SSU reviewed patients data, ZP reviewed patients data, AK reviewed patients records and performed the statistical analysis, TO reviewed patients records and performed the statistical analysis, AP performed the statistical analysis, EPC reviewed patients data; BJ exerted critical revision and supervision;

The manuscript has been seen and approved by all authors

## Supplementary Material

Additional file 1 **Thyroglobulin before and after 13-CRA RDT**. The data provided represent thyroglobulin concentration (ng/ml) before and after redifferentiation therapy in all 53 treated patients. Click here for file
