# Supplementary material for: 13-cis-retinoic acid re-differentiation therapy and recombinant human thyrotropin-aided radioiodine treatment of non-Functional metastatic thyroid cancer: a single-center, 53-patient phase 2 study
Source: Thyroid Res. 2009 Aug 1;2:8. doi: 10.1186/1756-6614-2-8 (PMC2739165; doi:10.1186/1756-6614-2-8)
Supplement: Additional file 1 — Thyroglobulin before and after 13-CRA RDT. The data provided represent thyroglobulin concentration (ng/ml) before and after redifferentiation therapy in all 53 treated patients. [file 1756-6614-2-8-S1.doc]

| patient | Tg day1 (ng/ml) | Tg day 42 (ng/ml) | Changes in Tg  level | 131-I uptake |
| --- | --- | --- | --- | --- |
| 1 | 228,0 | 273,0 | 20% | no |
| 2 | <0,2* | <0,2* | - | no |
| 3 | 4079,0 | 4668,0 | 14% | no |
| 4 | 230,0 | 350,5 | **52%** | **yes** |
| 5 | 34,0 | 39,0 | 15% | no |
| 6 | 60,0 | 65,0 | 8% | no |
| 7 | 19,5 | 35,0 | **79%** | no |
| 8 | 628,0 | 1305,0 | **108%** | no |
| 9 | 793,0 | 1115,0 | **41%** | no |
| 10 | 3,0 | 12,0 | **300%** | no |
| 11 | 36,4 | 69,2 | **90%** | no |
| 12 | 105,0 | 129,0 | 23% | no |
| 13 | 0,2* | 0,2* | - | no |
| 14 | 1091,0 | 1235,5 | 13% | no |
| 15 | <0,2* | <0,2* | - | no |
| 16 | 8,8 | 15,9 | **81**% | no |
| 17 | <0,2* | <0,2* | - | no |
| 18 | 0,5 | 0,3 | -40% | no |
| 19 | 356,0 | 589,0 | **65%** | no |
| 20 | 27,6 | 31,1 | 13% | no |
| 21 | nd | 177,9 | - | **yes** |
| 22 | 3830,0 | 3393,0 | -11% | no |
| 23 | nd | 160,0 | - | no |
| 24 | <0,2* | <0,2* | - | no |
| 25 | 3673,0 | 3137,0 | -15% | no |
| 26 | 55,7 | 66,0 | 18% | no |
| 27 | 7,5 | 16,4 | **119%** | no |
| 28 | 8,3 | 9,2 | 10% | **yes** |
| 29 | 124,0 | 107,5 | -13% | no |
| 30 | nd | 177,0 | - | no |
| 31 | 18,7 | 24,3 | 30% | no |
| 32 | <0,2* | <0,2* | - | **yes** |
| 33 | 12,0 | 10,7 | -11% | no |
| 34 | 41,2 | 62,5 | **52%** | no |
| 35 | 3938,0 | 3850,5 | -2% | **yes** |
| 36 | 1222,0 | 880,0 | -28% | no |
| 37 | 17,7 | 19,9 | 12% | no |
| 38 | 2492,0 | 2904,0 | 17% | **yes** |
| 39 | 8,0 | 7,7 | -4% | no |
| 40 | nd | 3627,0 | - | no |
| 41 | 280,0 | 330,0 | 18% | no |
| 42 | <0,2* | <0,2* | - | **yes** |
| 43 | 685,0 | 811,0 | 18% | no |
| 44 | 2685,0 | 3161,0 | 18% | **yes** |
| 45 | nd | 970,0 | - | no |
| 46 | 0,2 | 0,3 | **53%** | no |
| 47 | 84,9 | 81,3 | -4% | no |
| 48 | 79,0 | 132,0 | **67%** | no |
| 49 | 14,8 | 19,1 | 29% | no |
| 50 | 31,1 | 35,6 | 14% | no |
| 51 | 117,0 | 90,3 | -23% | **yes** |
| 52 | 25,4 | 44,4 | **75%** | no |
| 53 | nd | 4,2 | - | no |

- Tg recovery < 70%
